# CLOVE: classification of genomic fusions into structural variation events

**DOI:** 10.1186/s12859-017-1760-3

**Published:** 2017-07-20

**Authors:** Jan Schröder, Adrianto Wirawan, Bertil Schmidt, Anthony T. Papenfuss

**Affiliations:** 1 Bioinformatics Division, Walter and Eliza Hall Institute of Medical Research, 1G Royal Parade, Parkville, VIC 3052 Australia; 2 Department of Computing and Information Systems, University of Melbourne, Melbourne, VIC Australia; 3 Bioinformatics and Cancer Genomics, Peter MacCallum Cancer Centre, East Melbourne, VIC 3000 Australia; 4 Institut für Informatik, Johannes Gutenberg Universität Mainz, Mainz, Germany; 5 Department of Medical Biology, University of Melbourne, Melbourne, VIC 3010 Australia; 6 Sir Peter MacCallum Department of Oncology, University of Melbourne, Melbourne, VIC 3010 Australia; 7 Department of Mathematics and Statistics, University of Melbourne, Melbourne, VIC 3010 Australia

**Keywords:** Structural variations, Genomic rearrangements

## Abstract

**Background:**

A precise understanding of structural variants (SVs) in DNA is important in the study of cancer and population diversity. Many methods have been designed to identify SVs from DNA sequencing data. However, the problem remains challenging because existing approaches suffer from low sensitivity, precision, and positional accuracy. Furthermore, many existing tools only identify breakpoints, and so not collect related breakpoints and classify them as a particular type of SV. Due to the rapidly increasing usage of high throughput sequencing technologies in this area, there is an urgent need for algorithms that can accurately classify complex genomic rearrangements (involving more than one breakpoint or fusion).

**Results:**

We present CLOVE, an algorithm for integrating the results of multiple breakpoint or SV callers and classifying the results as a particular SV. CLOVE is based on a graph data structure that is created from the breakpoint information. The algorithm looks for patterns in the graph that are characteristic of more complex rearrangement types. CLOVE is able to integrate the results of multiple callers, producing a consensus call.

**Conclusions:**

We demonstrate using simulated and real data that re-classified SV calls produced by CLOVE improve on the raw call set of existing SV algorithms, particularly in terms of accuracy.

CLOVE is freely available from http://www.github.com/PapenfussLab.

**Electronic supplementary material:**

The online version of this article (doi:10.1186/s12859-017-1760-3) contains supplementary material, which is available to authorized users.

## Background

A structural variant (SV) is a rearrangement of the genome caused by at least two double strand DNA breaks followed by DNA repair. Typically, the term SV is used for events that are greater than 1 kb in size [1]. SVs include large insertions, inversions, balanced or unbalanced translocations, and amplifications and large deletions, collectively referred to as copy number variations (CNVs). A precise understanding of SVs is important in the study of population diversity, cancer [2–4] and other diseases (e.g. Charcot-Marie Tooth [5] and autism [6]).

The increasing usage of high throughput sequencing technologies has led to advances in the discovery and genotyping of structural variants in germline and somatic cells [7–9]. Consequently, a variety of methods have been developed to detect SVs from DNA sequencing data. Different approaches can be classified into four distinct categories: read depth (RD), discordant read pair (DR), split reads (SR), and de novo assembly (DN). RD methods involve counting reads in windows and segmenting the counts [10]. They identify only one class of structural variation (CNVs) and provide neither direct evidence for breakpoints, nor information about genomic organization. Their resolution and accuracy is dependent on sequencing coverage and window size, but is typically of the order of kilobases. Examples of RD methods include readDepth [10] and CNVnator [11]. DR methods use pair end-sequenced DNA fragments that span a breakpoint (typically in the un-sequenced region between the reads). These reads map anomalously or discordantly to the reference genome—further apart or closer together than expected based on the selected fragment size, to different chromosomes or with inverted orientation [12]. The signal of a rearrangement is a cluster of anomalous alignments. The resolution of DR methods is related to fragment size distribution and genome coverage. However, in general, single-nucleotide resolution is not possible with DR methods. BreakDancer [13] is an example of a DR method. SR methods rely on individual reads, which span the breakpoint and are capable of single-nucleotide resolution, although micro-homologies at breakpoint sites may introduce uncertainty. Examples of SR methods include Splitread [14] and Socrates [15]. DN methods use some form of assembly following other evidence to locate the locus of a rearrangement. DN methods typically provide single nucleotide resolution, but can be slow. Examples of DN methods are Cortex [16] and SOAPdenovo [17, 18].

Many tools utilize a hybrid approach that combines multiple lines of evidence to predict SVs. For example, Delly [19] and PRISM [20] use DR evidence and incorporate SR evidence through a targeted Smith-Waterman alignment. CNVer [12] uses DR and RD signals to identify potential copy number changes. CREST [21] uses SR then DN to directly map SVs at single nucleotide resolution, while SMUFIN [22] uses DN first and SR subsequently.

Another strategy is the “consensus” caller approach. For example, MetaSV [23] integrates a set of multiple tools into a pipeline. This approach aims to leverage the specific strengths of tools regarding different aspects of SV calling as well as confidence through agreement of multiple tools, and is therefore considered a meta-caller.

Nevertheless, the identification of SVs remains challenging. Existing methods suffer from a variety of issues relating to sensitivity and precision, positional accuracy and error profiles, and classification into one of the various types of SV. The majority of existing methods only identify breakpoints (a pair of connected breakends), also called genomic fusions (henceforth referred to as fusions), but do not classify the rearrangements further. Several SV callers are capable of limited classification of genomic events, such as insertions and deletions, but fail to classify more complex rearrangements. SVs may be simple (involving only a single fusion, such as an deletion) or more complex events (involving two or more fusions, such as a balanced translocation or inversion). Yang et al. [24] noted the lack of more complex events in the output of SV algorithms and introduced complex deletions, as well as inference of underlying DNA repair mechanisms. The study by Sudmant et al. [25] analysed a large cohort of human genomes for SVs including mobile element insertions. Another notable exception is the work by Escaramis et al. [26], which detects more complex events from aligned read data. However, the method is neither widely used nor cited, and failed to run on our data for purposes of comparison.

Here, we present a new method, CLOVE, which integrates calls from one or more breakpoint (or SV) detection methods and (re-)classifies the SV. Our method creates a graph data structure from the provided breakpoint information and then looks for patterns that are characteristic of more complex rearrangement types (e.g. balanced translocations). CLOVE is not another SV caller, but integrates multiple independent breakpoint predictions from other tools into a single, more accurate and potentially more complex event. This makes the output of these other tools more interpretable and increases the precision. A better-categorized output allows for better filtering or prioritization of specific events that are most relevant to the biological interpretation or experimental validation. CLOVE is the first meta-SV-caller that (i) can use any set of input (from current or future SV algorithms) to (ii) re-classify the data into more complex SVs than the original call sets.

## Methods

Our algorithm is capable of handling the breakpoint or SV calls produced by a variety of tools – in fact, it can handle multiple sets of fusion at the same time, benefiting from the increase in sensitivity in raw calls. CLOVE can be thought of as augmenting existing breakpoint callers. It allows for stratification of SVs into (i) sets of simple events that collectively present the signature of a complex event, and (ii) remaining simple events that pass or don’t pass a read depth check. CLOVE presents the stratified SVs in VCF format with additional SV types, statistics about the read depth of events, and the levels of support for events (i.e. how many SV callers support the SV). In the following, we distinguish between the notion of basic and complex SV types. Simple or basic SV types are those that are fully represented by a single fusion. Complex SVs contain more than one fusion.

### Basic SV types

**Fig. 1:**
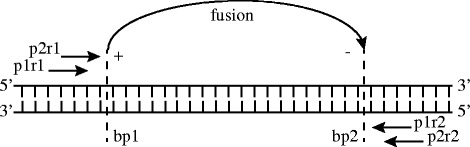
Example of a simple structural variant that illustrates how the signatures of fusions are defined. The *horizontal* structure in the middle represents the double-stranded DNA of a chromosome. Two read pairs are depicted as horizontal arrows mapping to the positive and negative strand of the DNA (the reads in the pairs are enumerated as pxry — pair x, read y). Assuming that the insert size of the two pairs are significantly above their expected value, an SV caller would call a deletion event from the two read pairs. The two *dashed vertical lines* indicate two breakends in the DNA. The breakends are connected by an arrow labelled “fusion”, which corresponds to the deletion event. The orientation signature of the fusion is indicated as “+” and “−“next to the breakends according to the mapping orientation of the reads that constitute the evidence to the fusion call

We introduce the following terminologies and conventions in order to discuss the different types of SVs that can be identified in sequencing data:

1. A *fusion* is a pair of loci that are adjacent in the donor genome but on different chromosomes or separated on the same chromosomes in the reference. The two separate locations of a fusion are referred to as breakends.
2. Each breakend has an orientation. We define the orientation to be “+” if the breakend occurs on the 3′ end of the fused region (on its right) in the reference, and “−” if it occurs at the 5’ end (on its left). If a fusion has DR support, the discordant reads flanking the breakend will be aligned “pointing” towards the break and the read strand will coincide with breakend orientation. Similarly, for a fusion with SR support, if the aligner maps the paired reads to the same side (left) of the breakend with the 3′ end of the fragment clipped, then the breakend has a “+” orientation. Figure 1 illustrates an example of a breakend in an intra-chromosomal event and DR support.
3. Based on the definitions above, we define a fusion as a pair of loci and directions: chromosome_1_ (chr_1_), position_1_, orientation_1_, chromosome_2_ (chr_2_), position_2_, orientation_2_.
4. If a fusion is an intra-chromosomal event, i.e. the event occurs in the same chromosome (chr_1_==chr_2_), by convention, we assume position_1_ ≤ position_2_.

Based on our definitions, a single fusion can be classified into eight categories (Table 1). Furthermore, we have assigned an event type to each category. These definitions are in line with the literature [19]. The labels are motivated by the fact that some of the more basic structural rearrangements in DNA bear the corresponding signatures. Note that the sub-classifications of inter-chromosomal translocation types require a chromosome ordering and assume chr_1_ < chr_2_. This definition is arbitrary but necessary to distinguish the two non-inverted events. Most SV callers that do provide event classification often follow the naming scheme presented in Table 1, or something similar. However, while the breakend signatures for these types are indeed consistent with the events that give them their names, these are not the only events that can cause such a signature. Overlooking this fact is often a source of classification error and confusion when interpreting fusions.

**Table 1:** Simple SV event labels defined by their breakend signature

Chromosomes|	Orientations		
	+		−
chr_1_ = chr_2_	+	Inversion type 1 (INV1)	Deletion type (DEL)
	−	Tandem duplication (TAN)	Inversion type 2 (INV2)
chr_1_ ≠ chr_2_	+	Inter-chromosomal inversion type 1 (INVTX1)	Inter-chromosomal translocation type 1 (ITX1)
	−	Inter-chromosomal translocation type 2 (ITX2)	Inter-chromosomal translocation type 2 (INVTX2)

Rows refer to the orientation of the first breakend and columns to the orientation of the second breakend. Simple events may be combined into complex event types; for example, an inversion (an inverted segment of DNA) is comprised of the two simple events INV1 and INV2

### Complex SV types

**Fig. 2:**
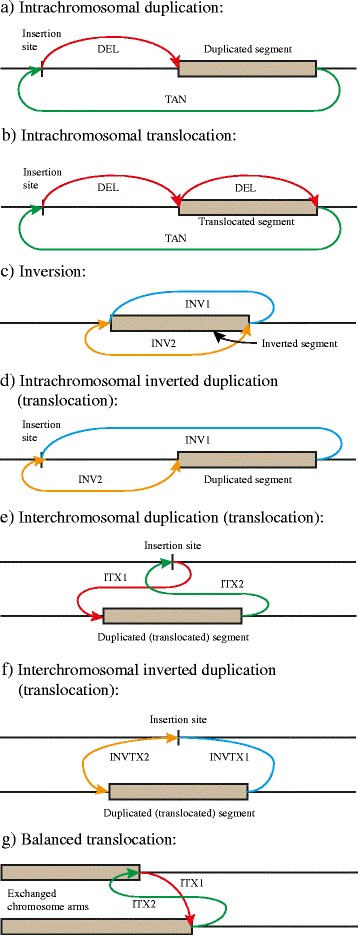
Rearrangement patterns (or signatures) for complex SVs. The connectors identify simple event types by color and arrowheads (for “−”orientation). **a**-**d** show events on the same chromosome, **e**-**g** show inter-chromosomal rearrangements. Intra-chromosomal duplications upstream of the insertion site are not shown in **a**) and are slightly different as they reverse the order of the deletion and tandem duplication events. Translocated rearrangements are not explicitly shown in **d**-**f** as they simply require a single deletion additional to the event types shown (see **b**))

The computation and output of more complex SVs, such as translocations or inversions, is typically not addressed by existing SV calling algorithms (or only parts thereof as discussed below). Figure 2a and b illustrate the rearrangement patterns created by interspersed duplications and translocations on a single chromosome. Both patterns consist of two to three lower order events of the tandem duplication or the deletion type. This observation motivates our approach to further classify SV calls: the SV output referring to deletions and duplications at sites where a more complex event has occurred is often confusing to the user, and not easily identifiable from the list of breakends. Note that duplications and translocations upstream of the insertion site have a slightly different pattern with the deletion event pointing at the insertion site. Figure 2c shows the signature of an inversion event. Some SV algorithms, e.g. CREST, can classify this type of rearrangement. Figure 2d shows the signature of inverted interspersed duplications on the same chromosome. Similar to the classifications in Fig. 2a-d, parts e-g show the signatures for complex inter-chromosomal events. The list of rearrangements presented here is probably not complete as far as all potential complex events go, but we believe covers the majority of relevant classes. The signal of complex events shown above is consistent with existing discussion of structural variants (such as by [26]). To our knowledge CLOVE is the first meta caller that categorises such a comprehensive list of SVs from fusion calls.

The complex SV events described above share a common principle: there is at least one locus in the rearrangement pattern where two or more breakpoints (or simple SV events) have a common breakend coordinate. This observation guides the design of CLOVE to discover rearrangement patterns in SV data.

With one exception, the patterns for each of the rearrangement events are also unique and specific. Due to the symmetric nature of the intra-chromosomal translocation event, it cannot be determined whether the block has moved as shown in Fig. 2b, or indeed the block marked with an “A” has translocated to just after the second deletion event. Notice that both alternatives result in the same string of DNA. We define the convention that the smaller of the two alternative blocks is considered to have translocated.

### Breakpoint graph construction and analysis

CLOVE has two major components, i.e. *complex* rearrangement pattern matching and read depth validation. Before these stages commence, the breakend calls are parsed into the algorithm. At the time of publication, the method supports outputs from Socrates, Delly, CREST, Gustaf [27], and BedPE (a standard format for fusion data). Furthermore, any SV caller can be re-classified, as long as it provides the sextet of information for each fusion introduced in Section 2.1. Any number of call sets can be input to CLOVE at the same time, which increases sensitivity if this adds non-redundant information.

The main internal data structure of CLOVE is a graph where nodes represent genomic coordinates (or coordinate intervals) and edges the fusions predicted by the SV callers. Fusions are added one at the time, and their coordinates are compared to the existing set of nodes. If a coordinate is within a user defined distance of a node, it is added to that node (modifying its coordinate interval if necessary). Otherwise, a new node (or pair of nodes) is created and added to the graph. Furthermore, one edge per fusion is added to the appropriate node(s). The edges (fusions) are labelled with one of the eight variant types introduced in Table 1. This data structure is similar, but not identical, to the breakpoint graph proposed by Bafna and Pevzner [28].

After the graph is constructed, CLOVE refines the existing edges in the graph. CLOVE scans the graph for redundant edges, i.e. edges between the same pair of nodes with identical SV type and merges them.

**Fig. 3:**
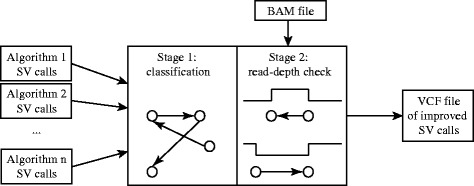
Outline of the workflow and key components of the CLOVE algorithm

The refined graph is subsequently analysed to identify complex rearrangement patterns. Afterwards, the classified SV events are validated using read depth information from the original short read dataset. Figure 3 illustrates the workflow of CLOVE to classify provided fusion data into improved SV calls.

The two main steps are now explained in more detail.

1. *Complex rearrangement pattern matching*: The initial classification step analyses the breakpoint graph in order to search for patterns described in Section 2.2. We iterate over the nodes in ascending coordinate order on each chromosome. If a node is adjacent with at least two edges, every edge-pair is investigated upon matching one of the complex SV rearrangement patterns (illustrated in Fig. 2). Intra-chromosomal duplication types are investigated first by matching tandem duplication and deletion types (or inversion1/2) accordingly. A further search for a deletion of the duplicated interval may then change the classification to a translocation. Any identified complex event is added to the graph and the (up to three) contributing events are removed from the graph. This step concludes once every node has been visited.
2. *Read depth validation*: The second stage of the classification procedure again traverses the graph in order to analyse the breakpoint consistency with the read depth within the according intervals. Deletion events are expected to include a relatively low read depth in-between the two breakends, while tandem duplications are expected to include an increased coverage. The read depth is established for each individual interval by querying the input BAM file. This value is compared to the expected coverage and standard deviation. A single deviation value is used in this step independent of the size of the interval. Although this is not the most rigorous approach, it works well in practice. The expected deviation is a user-supplied parameter – to be chosen reflecting the coverage spread of the analysed read data. It is key for this step to take place after the classification step. This way deletion types and tandem duplication type events have already been merged into higher order events that do not change the read depth (for example, translocations). Events that do not fulfil the expected coverage response are rejected from the classification, and instead output as events of secondary interest due to read depth inconsistency.

After the classification stages, CLOVE presents the results in a new output file in VCF format. The read depth is presented along with the levels of support for each event (informing about the number of tools that support an event and the number of events within the call sets).

Clove is implemented in *java* and makes use of the *htsjdk samtools* implementation to handle genomic intervals.

## Results and Discussion

To evaluate our method, we investigate results on simulated and real genomic data. The motivation behind the usage of simulated data is to allow full control over the sequence content, making an assessment of the sensitivity and precision of different tools possible. In the ideal case every simulated structural variant should be found and classified in the sequencing data, and no additional ones. Application to real data is important, but our knowledge about the variants present is incomplete.

We demonstrate that CLOVE is able to assign meaningful labels to rearrangement events. Furthermore, it increases the accuracy of the output of existing SV tools by removing a large proportion of false positive events. In the following two subsections, we compare the initial call set of different SV algorithms with the re-classified calls produced by CLOVE. For these comparisons, we investigate the sensitivity, precision, and accuracy statistics, which calls for the classic contingency tables of *true positives* (TP), *false positives* (FP), *false negatives* (FN) (and *true negatives*). Furthermore, we modify the standard approach as follows: we introduce a fifth contingency called *half-true-positives* (HTP). HTPs are defined as SV calls at the correct location but with the wrong event label (and potentially with too few fusions). This type allows us to make a fair comparison between the raw output (which often has few actual TPs) and the classified calls, without being overly harsh on existing methods – meaning, in the following HTPs are going to be counted as TPs for the calculations of recall, precision, and accuracy.

### Simulated data results

For this experiment, we use chromosomes 21 and 22 of the human reference (hg19) as the underlying genome. The simulation workflow is as follows:

1. The genetic material is divided into non-overlapping bins of length 500kbp. The binning strategy prevents events from overlapping and thus generating unresolvable complexities.
2. A random event type is chosen for each bin. The types we use here are deletion, tandem duplication, inversion, intra- and inter-chromosomal duplications, intra- and inter-chromosomal translocations, intra- and inter-chromosomal inverted duplications, and intra- and inter-chromosomal inverted translocations. For each event a random starting or insertion position is chosen within the bin, as well as a random length or duplication/translocation interval, respectively.
3. A new reference is generated that contains the above modifications.
4. Reads are simulated from the alternative reference (using SimSeq [29]) at a sequencing depth of 30× and 100 bp read length.
5. The reads are aligned to the original reference containing chromosomes 21 and 22 with Bowtie2 [30] in local alignment mode.
6. SVs are called with a variety of tools from the aligned read data. All tools are run with standard parameters.
7. CLOVE re-classifies the output of the SV methods generated in the previous step, and the results are compared to the list of variants created in Step 2). Clove is run without any parameters, except the coverage option, which is set to “-c 30 8” for this experiment.

**Fig. 4:**
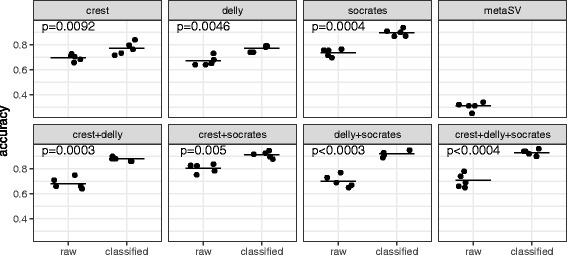
SV detection accuracy in simulated data before (raw) and after classification with CLOVE (classified). The scatter plots indicate performance for individual runs and the lines the average on the data. The significance of change in means is indicated by a *p*-value at the top of each panel (except for MetaSV, which is shown without CLOVE classification)

Steps 1–3 of the simulations is done by using tools introduced in [31]. This simulation workflow is repeated five times, generating new random events and reads each time. The classified SV calls of CLOVE (v0.14) are based on the outputs of Socrates (v1.13.1), Delly (v0.76) and CREST (v0.0.1) for each simulated dataset. Tests are conducted using both the output of each individual tool as input and outputs combined from two or all three tools as input. CLOVE results are then compared to the raw results of the corresponding SV algorithm(s) that have been used as input. Additionally, we include information about runs of MetaSV (using version 0.5.4 and calls provided by Pindel, Lumpy, and BreakDancer) by itself. The performance is measured in terms of sensitivity, precision, and accuracy. Figure 4 summarises the results for the accuracy metric. Note that Fig. 4 is based on calculations treating HTPs as TPs for the purpose of calculating accuracy – specifically, *accuracy* ≔ (*TP* + *HTP*)/(*TP* + *HTP* + *FP* + *FN*). The detailed individual results and performance per SV type are listed in the supplement (Additional file 1: Table S1). These results include the performance of Lumpy – not shown in Fig. 4. Lumpy performs with an average raw accuracy of 0.71, which improves to 0.82 when applying CLOVE. Interestingly, Lumpy’s performance is better than MetaSV’s, which uses its data as input.

The results show the following trends. First, there is increased accuracy due to the classification of events. Although some tools already call complex variants, there is not a single tool capable of the same level of accuracy as clove when run on multiple inputs. Consolidating the trend with more specific results in the supplementary material, we can conclude that this effect is caused by the increase in precision of re-classified breakpoints. Conversely, the recall is decreased after classification. Obviously, the recall cannot increase, since CLOVE does not create any additional events in the SV input – and a decrease is inevitable if an event does not pass the read depth check, or a complex event is missing an edge leaving the remaining one(s) to be discarded, whereas it is counted as HTP in the raw input. A summary of the sensitivity for each SV tool and CLOVE can be found seen in Additional file 2: Figure S1. It shows that there is no clear strengths and weaknesses for individual types (except for MetaSV struggling with anything but inversions, deletions, and tandem duplications), and that CLOVE’s sensitivity is competitive, but not (greatly) superior to that of individual tools.

Do these results allow us to answer the question if the genotyping is improving a given set of SV calls? This depends on the application. It is obvious that the precision is greatly improved, which is often desirable, sometimes critical to an experiment. This improvement effect is in fact stronger than the loss of sensitivity caused by the genotyping step, leading to overall increased accuracy. To demonstrate this, we can compare accuracy values before and after classification and apply a pairwise t-test. The change in means is statistically significant for each of the analysed methods. Finally, CLOVE allows usage of separate call sets of SVs in a single run of the classification algorithm. The graph cleaning steps discussed above facilitate the removal of redundant edges (events that have been called by two or more of the input methods). Taking the union of all input events, CLOVE has (potentially) more data to classify, which reduces the drop-off in recall after classification. We can indeed observe that the maximum average accuracy is achieved by CLOVE when using input from Crest, Delly, and Socrates at the same time. This is not quite the case for MetaSV, which shows overall low performance values (for both precision and sensitivity) despite having access to the output of multiple tools.

The simulated data and predicted SVs are available from http://bioinf.wehi.edu.au/clove/.

### Results for *Escherichia coli* K-12 strain

The utilized data set is a collection of single-end Illumina HiSeq 2500 reads from the K12 Beta 10 *E. coli* strain (http://www.ncbi.nlm.nih.gov/sra/SRX803011). The data is not affected by any specific conditions and thus should resemble the laboratory strain fairly closely. To introduce rearrangements into the sequence context, we compare the read data to a slightly distant reference strain: *E. coli* K12. This causes a number of relative genomic rearrangements in the donor genome on which we can test the effectiveness of CLOVE. Due to the single-ended nature of the data we are restricted to a subset of SV callers to evaluate for this experiment: Socrates and CREST. The experimental setup is as follows:

1. Align the reads to the K12 strain *E. coli* reference. For this purpose we use Bowtie2 (version 2.2.3) with the “--local” option. 6,254,124 reads align, amounting to an average haploid sequence coverage of 135×.
2. Run the SV calling algorithms. The tools produce a number of breakpoints, of which most are in concordance with each other. Again, we use standard parameters for the algorithms, except for the runs of Socrates where we set the minimum mapping quality to 0, as indicated below.
3. Establish which calls relate to real rearrangement events in the data and which relate to false positive predictions. This is a crucial step and the nature of the data allows us to make the required distinctions with high confidence. For once, the data is from a haploid genome without any expected changes except for those differences in strains (and maybe some mutations acquired in culture). Consequently, the allele frequencies of rearrangement events should be close to 100% for real events, except those involving multiple copies of the same genomic region. We use this property effectively by establishing the mutant allele frequency at each break and observing its ratio over reads that do not support the break at the same locus, but support the reference allele. Secondly, *E. coli* being a relatively small genome of relatively low complexity, we can manually check the predictions made by the SV callers upon credibility. With this we came across the issue of transposable elements in the *E. coli* reference: There are at least two groups of repetitive sequence in the reference that occur at 6 and 15 spots in the sequence. These elements actually drive the majority of the complex rearrangements that we observe in the donor genome. The problem that they present is mapping ambiguity (and therefore also rearrangement ambiguity). For example, the first such mobile element mentioned here is inserted into a new locus in the donor genome, but the question is, which of its six instances has been moved (or copied) to the new locus? For the sake of simplicity, we do not dwell on this problem too long, but assign a correct breakpoint call if any of the instances has been identified. It still poses a challenge to SV calling because copying an element to a new locus requires two fusions (see Section 2), and if not both fusions have been called for the same instance, CLOVE is not able to establish the correct event. A possible solution to this might be the creation of a new reference strain that consolidates all such repetitive elements into a single instance for the sake of mapping specificity. Unfortunately, this approach would also remove slight sequence differences that actually exist between the instances, and thus we refrained from this option.
4. Run CLOVE on the various input data for matched comparison to the raw output from SV calling algorithms. CLOVE is set to use the following coverage parameter for all input data sets: “-c 135 20”.

The following results are for the Socrates and Crest algorithms. Table 2 shows the results of SV discovery in the *E. coli* genome. We compare two different runs of Socrates where we select two different choices of the minimum mapping quality of re-aligned soft-clips. The motivation here is to demonstrate that CLOVE increases the precision of the re-classified output so much that it is beneficial to go for a more sensitive calling approach upfront. Table 2 demonstrates this gain in accuracy (from 0.6 on the standard parameters (M5) to 0.9 with heightened sensitivity (M0)). Unlike for the simulated data, the accuracy of Crest does not improve from the classification on this particular data set. However, precision is increased due to classification once again, but not by enough to make up for the drop in sensitivity. The reason for this strong decrease is the ambiguity in mapping location as highlighted above. The best results on this data are achieved by combining the available results from both algorithms (Crest plus Socrates), as was the case on simulated data. While the raw data suffers from high numbers of false positives after combination, the classified data has perfect precision making it the most useful set of SV events to investigate.

**Table 2:** Results for SV recovery from the output of Socrates and Crest before (^R^) and after (^C^) classification with CLOVE in real sequencing data

Organism	Tool	Data	TP	HTP	FP	FN	Sn (95% CI)	Pr (95% CI)	Acc (95% CI)
Ecoli (SRX803011)	Socrates	M5^R^	8	7	33	6	0.71 (.50,.86)	0.31 (.20,.45)	0.28 (.18,.40)
		M5^C^	12	0	0	8	0.60 (.39,.78)	1.00 (.76,1.0)	0.60 (.39,.78)
		M0^R^	8	13	49	0	1.00 (.85,1.0)	0.30 (.21,.42)	0.30 (.21,.42)
		M0^C^	17	2	0	2	0.90 (.71,.97)	1.00 (.83,1.0)	0.90 (.71,.97)
	Crest	R	7	7	4	6	0.70 (.48,.85)	0.77 (.55,.91)	0.58 (.39,.76)
		C	10	0	0	10	0.50 (.30,.70)	1.00 (.72,1.0)	0.50 (.30,.70)
	Socrates + Crest	R	8	13	50	0	1.00 (.85,1.0)	0.30 (.20,.41)	0.30 (.20,.41)
		C	17	3	0	1	0.95 (.77,1.0)	1.00 (.84,1.0)	0.95 (.77,1.0)
Human (NA12878)	Delly	R	1447	0	6818	1932	0.43 (.41,.44)	0.18 (.17,.18)	0.14 (.14.15)
		C	1437	0	0	1942	0.43 (.41,.44)	1.00 (1.0,1.0)	0.43 (.41,.44)
	Socrates	R	900	0	3781	2361	0.28 (.26,.29)	0.19 (.18,.20)	0.13 (.12.14)
		C	894	0	0	2403	0.27 (.26,.29)	1.00 (1.0,1.0)	0.27 (.26,.29)
	Delly + Socrates	R	1819	0	10,394	1464	0.55 (.54,.57)	0.14 (.14,.16)	0.13 (.13,.14)
		C	1816	0	0	1493	0.55 (.53,.57)	1.00 (1.0,1.0)	0.55 (.53,.57)

Columns refer to true positive events (TP), half true positives (HTP), false positives (FP), false negatives (FN), sensitivity/recall (Sn), precision (Pr), and accuracy (Acc). Confidence intervals calculated through binconf in R are supplied for the latter three columns

### Results for NA12878

Finally, we demonstrate how CLOVE performs on the widely studied NA12878 cell line (Illumina sequencing data to 50× coverage with 100 bp PE reads; ENA accession: ERA172924). As there are previously validated deletion calls [32], we have a truth set for this type of variant to compare to. We subset the output of Delly and Socrates to those of deletion calls only and then run CLOVE on the data. Similar to our observations in Sections 3.1 and 3.2, the precision is greatly increased while the sensitivity suffers slightly. More specifically, the details of the experiment can be seen in Table 2, and highlight the gain in accuracy by up to 0.42.

When using the entire set of fusions produced by Delly and Socrates, CLOVE is able to classify complex events from the data. When filtering events which insertion point (where applicable) is in a repetitive region, there is a total of 1922 events in called for the cell line. The variants are deletions (1338), tandem duplications (332), inter-chromosomal (/inverted) duplications (104/87), interspersed (/inverted) duplications (35/12), and inversions (13). We randomly selected 4 such events (an inversion, duplication, inter-chromosomal duplication and inverted duplication) and were able to demonstrate that for each of these the mapping of Pacbio long read data [33] could be improved when aligning to an alternative reference containing the predicted reference (we consider a variant validated if a Pacbio read maps across the SV including all fusions, and the alignment has a higher score than in the original reference). For more detail see the Additional file 3: Data S1.

## Future work

CLOVE has been developed for and experimented with on germline data. In other types of data, such as cancer genomes, types of variants other than those currently identified by CLOVE may be present. For example, there are large scale chromosomal rearrangements, such as chromothripsis and breakage fusion bridge, common among some cancers. Further, compound events, where two or more of the events described in this work co-occur at the same locus, have also been observed in tumour genomes. It would be desirable to add rules and functionality to CLOVE to deal with such events. However, this is met with technical challenges of potentially incomplete fusion signatures (false negative calls), and an explosion of the rule set (for all potential compound events).

## Conclusions

We have presented a new method for classifying complex rearrangement from breakpoint calls generated by different algorithms. We have demonstrated CLOVE’s ability to improve the output of standard SV methods by highlighting biologically relevant features, prioritizing, enhancing the precision of the calls, and generally improving accuracy. CLOVE’s independence of the input algorithm makes it a flexible tool to utilize in any SV calling pipeline. Its ability to process joint inputs from multiple methods is an attractive feature, which often leads to even better rearrangement classification, as has been indicated by our results. CLOVE is the first meta-caller that can use the input of any SV algorithm (provided it outputs sufficient information).

## Additional files

Additional file 1: Figure S1. Description of data: Sensitivity of individual tools and one run on CLOVE for different event types. Sensitivity is measured including half true positives (wrong event type). Events are considered recalled if any one of its fusions is found in the output. (PDF 9 kb)

Additional file 2: Table S1. Description of data: Detailed results of simulated data analysis. The spreadsheet shows runs of the tested structural variant tools as well as CLOVE re-classified results by variant type and for the individual runs of simulated data. (XLSX 139 kb)

Additional file 3: Data S1. Description of data: VCF file of variant calls of CLOVE on the NA12878 genome. (VCF 271 kb)

## Abbreviations

CNV: Copy number variation
DR: Discordant read pair
FN: False negative
FP: False positive
HTP: Half true positive
RD: Read depth
SR: Split read
SV: Structural variation
TP: True positive
