# Supplementary figures and images for: CLOVE: classification of genomic fusions into structural variation events
Source: BMC Bioinformatics. 2017 Jul 20;18:346. doi: 10.1186/s12859-017-1760-3 (PMC5520322; doi:10.1186/s12859-017-1760-3)

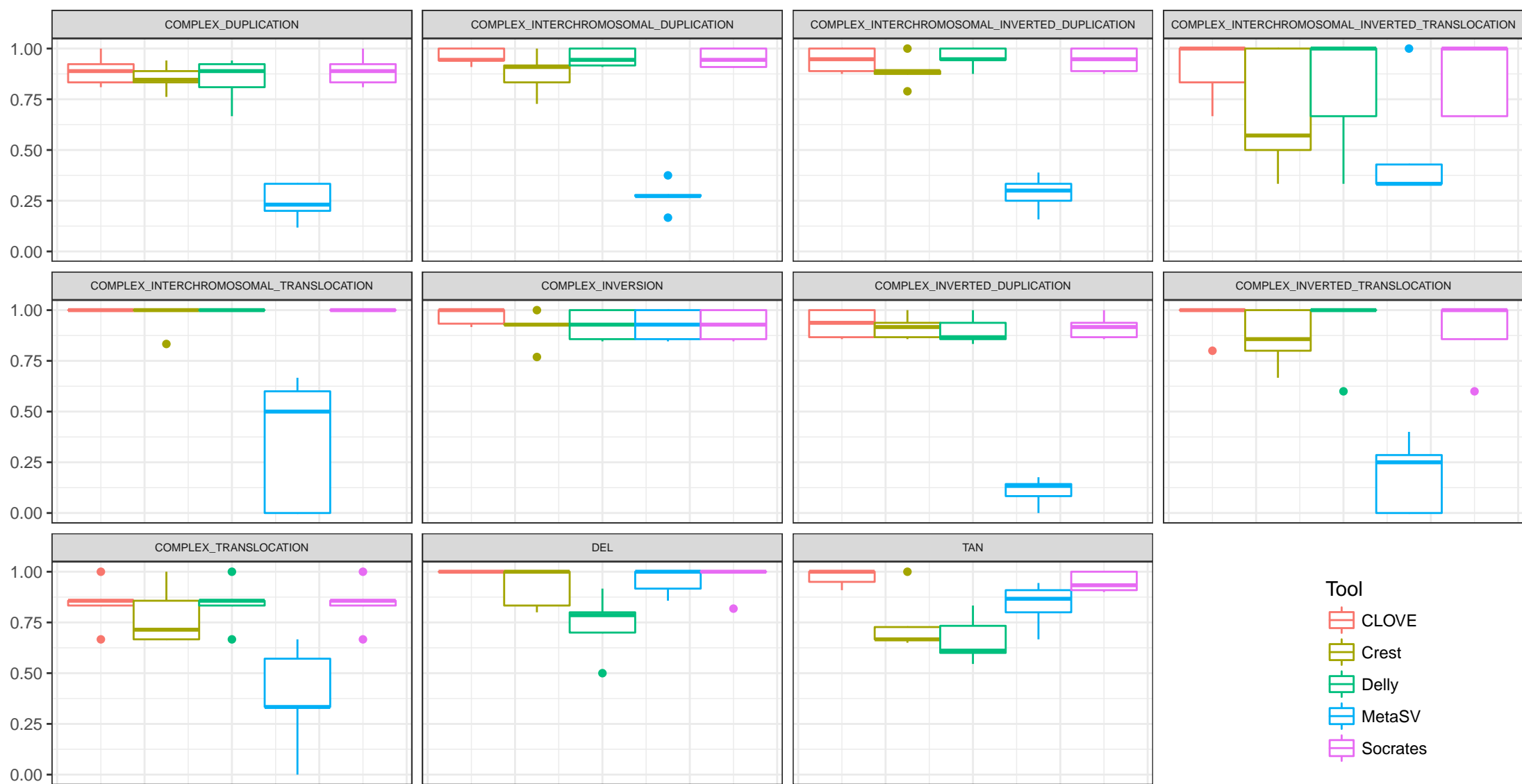

Supplement: Supplementary file 1 — Description of data: Sensitivity of individual tools and one run on CLOVE for different event types. Sensitivity is measured including half true positives (wrong event type). Events are considered recalled if any one of its fusions is found in the output. (PDF 9 kb) [file 12859_2017_1760_MOESM1_ESM.pdf]
